# Dental Caries in 7–17-Year-Old Children in Moscow: A Clinical and a Questionnaire Study

**DOI:** 10.3290/j.ohpd.a43358

**Published:** 2020-07-04

**Authors:** Irina Kuzmina, Kim Rud Ekstrand, Vibeke Qvist, Liubov Demuria, Azam Bakhshandeh

**Affiliations:** a Professor, Preventive Dentistry Department, Moscow State University of Medicine and Dentistry, Moscow, Russia. Supervisor, contributed to conception, design, data acquisition, analysis, and interpretation, drafted and critically revised the manuscript.; b Professor, Section of Cariology and Endodontics, Department of Odontology, Faculty of Health and Medical Sciences, University of Copenhagen, Copen­hagen, Denmark. Contributed to data analyses and interpretation, drafted and critically revised the manuscript.; c Associate Professor, Section of Cariology and Endodontics, Department of Odontology, Faculty of Health and Medical Sciences, University of Copen­hagen, Copenhagen, Denmark. Contributed to data analyses and interpretation, drafted and critically revised the manuscript.; d PhD Student, Preventive Dentistry Department, Moscow State University of Medicine and Dentistry, Moscow, Russia. Principal investigator, contributed to design, data acquisition and interpretation, drafted and critically revised the manuscript.; e Associate Professor, Section of Cariology and Endodontics, Department of Odontology, Faculty of Health and Medical Sciences, University of Copenhagen, Copenhagen, Denmark. Contributed to data analyses and interpretation, drafted and critically revised the manuscript.

**Keywords:** caries, clinical research, epidemiology, oral health, paediatric dentistry

## Abstract

**Purpose:**

Sparse data is available concerning the distribution of decayed, extracted, filled/decayed, missing, filled tooth surfaces (defs/DMFS) and the impact of influencing risk factors in Moscow. We thus aimed to measure caries experience and to estimate its associations with relevant risk factors in schoolchildren.

**Materials and Methods:**

Data was obtained from 1004 schoolchildren aged 7–17. The clinical examination included the status of dental plaque, gingival bleeding and caries experience; defs/DMFS. The questionnaire was introduced to the children/parents, in order to measure socioeconomic and behavioural variables. The Fisher Exact test and chi-squared test were used to assess statistical significance of the distribution of the variables among groups. Bivariate and general estimating equations (GEE) analyses were applied to estimate the relative effect of the independent variables on the outcomes defined as median defs and median DMFS.

**Results:**

Of 95 participants, 79 (83%) were male and 37 (39%) were homeless. Statistically significant difference between the groups was observed in 6–12-mm periodontal pocket depths (p <0.05), as were differences in oral mucosal changes (p <0.001). Statistically significantly lower proportions were observed in the cannabis group for Mob G:0 and Mob G:1 and Furcation G:1 compared to the CNSS and opiate groups; the proportion of Furcation G:0 was significantly lower in the alcohol group compared to the cannabis group. Analysis of variance (ANOVA) revealed statistically significant between-group differences in age, number of years of substance use disorder, number of teeth, and decayed, missing and filled teeth (DMFT). When controlling for age and gender, substance type was found to be a statistically significant predictor of number of teeth (B = –4.4; 95% CI: –8.1 to -0.38; p = 0.03) and DMFT (B = 2.1; 95% CI: 0.86 to 3.3; p = 0.001).

**Conclusions:**

Clinical, socioeconomic and behavioural determinants were identified to influence caries in primary and permanent dentition in schoolchildren in Moscow. The findings might provide a reliable basis for improvements and education programmes in oral health promotion for children and adolescents.

Dental care for children in Russia is delivered by the public dental health service (PDHS). Even though there have been a lot of structural changes in PDHS during the last 20 years, sparse data regarding defs, DMFS (decayed, extracted, filled, decayed, missing, filled tooth surfaces) and influencing factors on dental caries in form of abstracts is available. From 1998 to 2008 and in 2013, the mean DMFT (decayed, missing, filled teeth) had dropped further to 2.0; yet with some deviation within the Moscow districts.^11^ The mean deft for 6-year-olds in Moscow was 4.7 in 2012.^11^ According to data from the National Oral Health Survey, the mean deft of 6-year-olds in Moscow increased from 3.8 in 1998 to 4.0 in 2008.^14^ However, published data from 1993, from Solntsevsky district in Moscow,^13^ showed a mean deft of 5.6 for 6-year-olds, indicating a small reduction in the prevalence of caries in the primary dentition in Moscow within the last 20 years.

During the last decades, the prevalence of dental caries has declined in several industrialised countries.^5,7^ The Nordic countries have shown a marked reduction in dental caries since the 1980s, due to the establishment of public health programmes which incorporate disease prevention and health promotion.^6^ Factors such as effective use of fluoride, better self-care and control of risk factors through family engagement and well-functioning cooperation with schools have also influenced the improvement of oral health. However, the timeframes for decline differed among the Scandinavian countries due to variations in decisions concerning preventive strategies between the different dental professionals in each country, and between the countries.^3,9^

Information about the severity of caries experience among children is needed in Moscow. Moreover, no published information exists for clarifying the statistically significant effect of key caries risk factors. The primary aims for the present study were to determine the level of defs in the primary dentition and the level of DMFS in the permanent dentition among the target population. The secondary aims were to find out which kind of variables influenced the median defs and the median DMFS in the target population, using the World Health Organization (WHO) questionnaire data.

## MATERIALS AND METHODS

The study comprised a clinical examination and a questionnaire investigation. Both were conducted in the Central District of Moscow between January and March 2014. Ten schools accepted the invitation to participate in the study. Approximately 4000 children and adolescents aged 7–17 attended the ten public schools in the district. All ten schools had dental clinics: seven fully equipped for simple treatment procedures and three partly equipped; mainly used for dental check-ups. If treatment were needed, the children were referred to the public dental clinic for all kinds of treatments. All children who came to these schools for dental check-ups or emergency treatment were invited to take part in the study. All participants, school principals, teachers and parents were informed about the objectives of the study. Parents signed an inform consent paper. Informed consent was obtained from parents and children prior to the study, which was approved by the Ethical Committee of Moscow State University of Medicine and Dentistry.

### Clinical Study

Calibration of four dentists was performed by the first author (IK), at the university clinic of the Moscow State University of Medicine and Dentistry (MSUMD). The dental examination included the status of dental plaque, gingival bleeding and caries experience (defs/DMFS). Training and calibration in caries, plaque and gingival status evaluation was done before the study. Plaque was scored, occlusally on tooth 46 (or 85 in case 46 was absent), facially on tooth 22 (or 62 in case 22 was absent), and lingually on tooth 36 (or 75 in case 36 was absent). Plaque was scored as not visible, thin plaque (slight evidence of plaque) or thick plaque (easily detectable plaque).^2^ If plaque was not easily visible, a probe was used. The presence of plaque on the probe was recorded as thin plaque. Gingival status was scored on the buccal surfaces on tooth 16 (or 55), 12 (or 52), 32 (or 72), and 36 (or 75) as sound, bleeding on gentle probing, or bleeding after air blowing using modified definitions from Löe (1967).^15^ Caries registration was performed visually under dental unit light, but without cleaning and drying, and only at the traditionally cavitated level.^4^ Restored teeth and teeth lost due to caries were also recorded.

### Questionnaire Study

The questionnaire was based on Annex 8 from the World Oral Health Questionnaire scheme for children.^18^ All questions were translated into Russian. The scheme was used after removing the question concerning use of tobacco among the young children. The questions included personal information (age and gender), oral health status, oral health habits, eating and drinking habits, and education level of the parents (Table 1). The questionnaire was validated and pretested to avoid potential misunderstanding by respondents. All children and adolescents received a structured questionnaire for them to complete.

**Table 1:** Baseline information about clinical examination and answers to questionnaire in primary dentition (7–12 years old) and permanent dentition (7–17 years old)

Variable	Threshold	Permanent dentition (DMFS)						Primary dentition (defs)					
		≤ median defs		> median defs		Total		≤ median DMFS		> median DMFS		Total	
n	%	n	%	n	%	n	%	n	%	n	%
Age	7 years old	16	53%	14	47%	30	100%	26	87%	4	13%	30	100%
	8 years old	29	49%	30	51%	59	100%	40	68%	19	32%	59	100%
	9 years old	36	47%	41	53%	77	100%	36	47%	41	53%	77	100%
	10 years old	40	49%	41	51%	81	100%	59	73%	22	27%	81	100%
	11 years old	71	59%	50	41%	121	100%	76	63%	45	37%	121	100%
	12 years old	93	78%	26	22%	119	100%	58	47%	65	53%	123	100%
	13 years old	–	–	–	–	–	–	66	64%	37	36%	103	100%
	14 years old	–	–	–	–	–	–	36	47%	40	53%	76	100%
	15 years old	–	–	–	–	–	–	47	49%	49	51%	96	100%
	16 years old	–	–	–	–	–	–	73	48%	79	52%	152	100%
	17 years old	–	–	–	–	–	–	55	64%	31	36%	86	100%
	Total	285	59%	202	41%	487	100%	572	57%	432	43%	1004	100%
Gender	Girl	156	66%	81	34%	237	49%	312	58%	225	42%	537	53%
	Boy	129	52%	121	48%	250	51%	260	56%	207	44%	467	47%
	Total	285	59%	202	41%	487	100%	572	57%	432	43%	1004	100%
Plaque	No plaque	169	63%	99	37%	268	55%	297	62%	185	38%	482	48%
	Thin plaque	109	56%	85	44%	194	40%	252	53%	225	47%	477	48%
	Thick plaque	7	28%	18	72%	25	5%	23	51%	22	49%	45	4%
	Total	285	59%	202	41%	487	100%	572	57%	432	43%	1004	100%
Gingival bleeding	No bleeding	218	56%	172	44%	390	80%	429	63%	255	37%	684	68%
	Bleeding on probing	57	70%	25	30%	82	17%	124	43%	165	57%	289	29%
	Bleeding after air blowing	10	67%	5	33%	15	3%	19	61%	12	39%	31	3%
	Total	285	59%	202	41%	487	100%	572	57%	432	43%	1004	100%
Health of teeth?	Excellent/very good	66	52%	62	48%	128	26%	164	64%	92	36%	256	25%
	Others	219	61%	140	39%	359	74%	408	55%	340	45%	748	75%
	Total	285	59%	202	41%	487	100%	572	57%	432	43%	1004	100%
Health of gums?	Excellent/very good	119	62%	72	38%	191	39%	261	59%	179	41%	440	44%
	Others	165	56%	130	44%	295	61%	310	55%	253	45%	563	56%
	Total	285	59%	202	41%	486	100%	285	59%	202	41%	1003	100%
Toothache?	Often/occasionally	56	49%	59	51%	115	24%	120	52%	111	48%	231	23%
	Others	226	62%	139	38%	365	76%	448	58%	318	42%	766	77%
	Total	282	59%	198	41%	480	100%	568	57%	429	43%	997	100%
Dentist visit last 12 months?	Up to several times	227	58%	164	42%	391	82%	468	56%	369	44%	837	84%
	None	54	64%	30	36%	84	18%	97	63%	58	37%	155	16%
	Total	281	59%	194	41%	475	100%	565	57%	427	43%	992	100%
Reason for dentist visit?	Pain or troubles	26	52%	24	48%	50	11%	42	53%	38	48%	80	9%
	Others	234	59%	160	41%	394	89%	496	58%	359	42%	855	91%
	Total	260	59%	184	41%	444	100%	538	58%	397	42%	935	100%
How often do you clean your teeth?	< once daily	18	44%	23	56%	41	8%	51	52%	48	48%	99	10%
	Up to several times daily	267	60%	179	40%	446	92%	521	58%	384	42%	905	90%
	Total	285	59%	202	41%	487	100%	572	57%	432	43%	1004	100%
Toothbrush	Yes	285	59%	202	41%	487	100%	571	57%	431	43%	1002	100%
	No	–	–	–	–	–	–	1	50%	1	50%	2	–
	Total	285	59%	202	41%	487	100%	572	57%	432	43%	1004	100%
Toothpicks and/or dental floss	Yes	113	59%	80	41%	193	40%	248	63%	147	37%	395	39%
	No	172	59%	122	41%	294	60%	324	53%	285	47%	609	61%
	Total	285	59%	202	41%	487	100%	572	57%	432	43%	1004	100%
Charcoal	Yes	14	61%	9	39%	23	5%	23	56%	18	44%	41	4%
	No	271	58%	193	42%	464	95%	549	57%	414	43%	963	96%
	Total	285	59%	202	41%	487	100%	572	57%	432	43%	1004	100%
Chewstick/ miswak	Yes	2	40%	3	60%	5	1%	6	55%	5	45%	11	1%
	No	283	59%	199	41%	482	99%	565	57%	427	43%	992	99%
	Total	285	59%	202	41%	487	100%	572	57%	432	43%	1003	100%
Do you use toothpaste with fluoride?	Yes	44	53%	39	47%	83	18%	140	61%	89	39%	229	24%
	No	223	60%	147	40%	370	82%	406	55%	335	45%	741	76%
	Total	267	59%	186	41%	453	100%	546	56%	424	44%	970	100%
Not satisfied with appearance of my teeth?	Yes	42	48%	46	52%	88	19%	84	57%	64	43%	148	15%
	No	237	61%	150	39%	387	81%	480	0.57	364	0.431	844	85%
	Total	279	59%	196	41%	475	100%	564	0.57	428	0.431	992	100%
Avoid smiling because of my teeth?	Yes	30	64%	17	36%	47	10%	43	61%	28	39%	71	7%
	No	248	58%	176	42%	424	90%	519	57%	398	43%	917	93%
	Total	278	59%	193	41%	471	100%	562	57%	426	43%	988	100%
Children make fun of my teeth	Yes	7	58%	5	42%	12	3%	16	62%	10	38%	26	3%
	No	270	59%	184	41%	454	97%	545	57%	416	43%	961	97%
	Total	277	59%	189	41%	466	100%	561	57%	426	43%	987	100%
Don’t go to school because of toothache or discomfort	Yes	10	59%	7	41%	17	4%	16	62%	10	38%	26	3%
	No	268	59%	185	41%	453	96%	545	57%	416	43%	961	97%
	Total	278	59%	192	41%	470	100%	561	57%	426	43%	987	100%
Difficulty biting hard food	Yes	14	64%	8	36%	22	5%	29	64%	16	36%	45	5%
	No	265	59%	184	41%	449	95%	532	56%	411	44%	943	95%
	Total	279	59%	192	41%	471	100%	561	57%	427	43%	988	100%
Difficulty in chewing	Yes	6	60%	4	40%	10	2%	16	73%	6	27%	22	2%
	No	270	59%	187	41%	457	98%	544	57%	418	43%	962	98%
	Total	276	59%	191	41%	467	100%	560	57%	424	43%	984	100%
Fresh fruit	Yes	239	57%	180	43%	419	86%	480	57%	364	43%	844	84%
	No	46	68%	22	32%	68	26%	92	58%	68	43%	160	27%
	Total	285	59%	202	41%	487	100%	572	57%	432	43%	1004	100%
Biscuits, cakes, cream cakes, sweet pies, buns, etc.	Yes	149	57%	114	43%	263	54%	319	53%	279	47%	598	60%
	No	136	61%	88	39%	224	46%	253	62%	153	38%	406	40%
	Total	285	59%	202	41%	487	100%	572	57%	432	43%	1004	100%
Lemonade, Coca Cola, other soft drinks	Yes	85	55%	69	45%	154	32%	215	50%	213	50%	428	43%
	No	200	60%	133	40%	333	68%	357	62%	219	38%	576	57%
	Total	285	59%	202	41%	487	100%	572	57%	432	43%	1004	100%
Jam/honey	Yes	99	55%	80	45%	179	37%	222	51%	210	49%	432	43%
	No	186	60%	122	40%	308	63%	350	61%	222	39%	572	57%
	Total	285	59%	202	41%	487	100%	572	57%	432	43%	1004	100%
Chewing gum with sugar	Yes	101	56%	78	44%	179	37%	236	57%	178	43%	414	41%
	No	184	60%	124	40%	308	63%	336	57%	254	43%	590	59%
	Total	285	59%	202	41%	487	100%	572	57%	432	43%	1004	100%
Sweets/candy	Yes	107	54%	92	46%	199	41%	242	56%	188	44%	430	43%
	No	178	62%	110	38%	288	59%	330	57%	244	43%	574	57%
	Total	285	59%	202	41%	487	100%	572	57%	432	43%	1004	100%
Milk with sugar	Yes	75	51%	71	49%	146	30%	162	55%	135	45%	297	30%
	No	210	62%	131	38%	341	70%	410	58%	297	42%	707	70%
	Total	285	59%	202	41%	487	100%	572	57%	432	43%	1004	100%
Tea with sugar	Yes	163	55%	132	45%	295	61%	300	60%	200	40%	500	50%
	No	122	64%	70	36%	192	39%	272	54%	232	46%	504	50%
	Total	285	59%	202	41%	487	100%	572	57%	432	43%	1004	100%
Coffee with sugar	Yes	38	55%	31	45%	69	14%	83	55%	69	45%	152	15%
	No	247	59%	171	41%	418	86%	489	57%	363	43%	852	85%
	Total	285	59%	202	41%	487	100%	572	57%	432	43%	1004	100%
Education of your father	High school or less	12	63%	7	37%	19	5%	20	54%	17	46%	37	4%
	College/University	221	58%	160	42%	381	95%	456	56%	352	44%	808	96%
	Total	233	58%	167	42%	400	100%	476	56%	369	44%	845	100%
Education of your mother	High school or less	9	56%	7	44%	16	4%	15	52%	14	48%	29	3%
	College/University	238	59%	168	41%	406	96%	502	57%	378	43%	880	97%
	Total	247	59%	175	41%	422	100%	517	57%	392	43%	909	100%


#### Statistical Methods

Intra- and interexaminer reliabilities on defs and DMFS were calculated using unweighted kappa. The statistical analyses were performed separately for primary dentition (7–12-year-olds) and permanent dentition (7–17-year-olds). For the analyses, the children with primary dentition were grouped into two age groups (7–10 and 11–12) and the children with permanent dentition were divided into four age groups (7–10/11–12/13–14/15–17). Initially, it was tested whether the dependent variable, expressed as defs/DMFS, followed a normal distribution within the different age groups, which was not the case. We were left to use either defs/DMFS = 0 and defs/DMFS >0, or defs/DMFS below and above the median, as statistics in this investigation. Since very few participants had a defs = 0 and many had a DMFS = 0, particular among the younger age groups, we used the median values for each age group as the final statistics concerning the dependent outcome variables. Median defs and median DMFS were calculated for each age and dichotomised to < or > the median defs and median DMFS. The Fisher’s exact test and chi-squared test were used to assess the statistical significance of the associations of the independent variables on the outcomes median defs and median DMFS. The generalised estimation equation (GEE) was used to estimate the relative effect of the independent variables on the outcomes.^1^ In all tests the level of statistical significance was set at 0.05. Data were analysed in IBM-SPSS software.^8^

### RESULTS

In January 2014, 20 patients (7–17-year-olds) were examined twice with an interval of 2 days. Substantial agreement was found between interexaminer (Kappa: 0.74–0.76) and intraexaminer (Kappa: 0.77–0.81) reproducibility for the clinical examination concerning defs and DMFS.

A total of 1004 out of 4000 children aged 7–17 were included in the study; 47% of the children were boys and 53% were girls (Table 1). The distribution of defs and DMFS on each age is shown in Table 2. Of the 1004 children involved, 487 (237 girls/250 boys) had mixed dentition, so that data for these 487 children were used to assess the associations between median defs and risk factors in the primary dentition.

**Table 2:** **Table 2** Distribution of min, max, mean and median values for defs and DMFS in relation to age of the patients

Age	Primary dentition defs				Permanent dentition DMFS				n
	Min	Max	Mean	Median	Min	Max	Mean	Median	
7 years old	1	51	9.53	4.0	0	3	0.20	0.0	30
8 years old	0	40	13.58	14.0	0	4	0.63	0.0	59
9 years old	0	46	12.91	11.0	0	7	1.42	1.0	77
10 years old	0	44	12.91	11.0	0	6	0.56	0.0	81
11 years old	0	20	3.01	0.0	0	12	1.26	0.0	121
12 years old	0	50	1.79	0.0	0	14	2.42	2.0	123
13 years old	0	4	0.05	0.0	0	8	1.56	0.0	103
14 years old	0	1	0.01	0.0	0	20	3.80	2.0	76
15 years old	0	0	0.0	0.0	0	16	4.05	3.0	96
16 years old	0	0	0.0	0.0	0	16	3.89	3.0	152
17 years old	0	0	0.0	0.0	0	14	2.50	0.0	86


Information from the questionnaire indicated that 49% of the children had attended a dental clinic once or twice during the last 12 months, while the rest had dental visits ≥ 3 times. The majority (85%) of the dental visits concerned treatment/treatment follow-up or routine check-ups, and 9% were related to pain in the teeth/gingiva/mouth with no differences between the genders (p >0.05).

For the 7–10-year-olds (n = 247) the median defs was 6.0 (mean: 10.7, range: 0–51) and the median DMFS was 0 (mean: 0.8, range: 0–7). For the 11–12-year-olds (n = 240) the median defs was 0 (mean: 2.4, range: 0–40) and the median DMFS was 1.0 (mean: 1.7, range: 0–12). For the 13–14-year-olds (n = 183), the median DMFS was 1.0 (mean: 2.7, range: 0–20). For the 15–17-year-olds (n = 334), median DMFS was 2.0 (mean: 3.6, range: 0–16). The major components of defs among 7–10-year-olds were decayed and filled surfaces, while exfoliated teeth made up most of the index in the older group.

Thin plaque was recorded for 48% and the presences of thick plaque for 4% of the 1004 participants (Table 1). The presence of plaque (thin and thick) was highest among the 13–14-year-olds (62%) and lowest among the 7–10-year-olds (37%), (p < 0.001). Gingival bleeding on probing and/or after air blowing at the recorded teeth was registered for 32% of the 1004 participants (Table 1). Further analyses disclosed that the presence of gingival bleeding was lowest among the 7–10-year-olds (11%) and highest among 15–17-year-olds (46%) (p < 0.001).

Table 1 also provides data from all questions dichotomised into ‘yes’ or ‘no’ answers, expressed for the 487 children with primary teeth and the entire target group of 1004 children and adolescents.

### Bi- and Multivariate Analyses, Primary Dentition (7–12-Year-Olds, n = 487)

In the bivariate analyses, the independent variables of age, gender, plaque, toothache, satisfaction with teeth’s appearance, and intake of milk with sugar significantly influenced the median defs (p ≤ 0.045). The following multivariate GEE analyses revealed that the median defs was higher in younger age group (OR = 1.2, CI = 1.1–1.3, p <0.001), lower in girls (OR = 0.9, CI = 0.8–0.9, p <0.001), and children with no plaque (OR = 0.7, CI = 0.6–0.9, p <0.001) or thin plaque (OR = 0.8, CI = 0.7–1.0, p = 0.002).

### Bi- and Multivariate Analyses, Permanent Dentition (7–17-Year-Olds, n = 1004)

In the bivariate analyses, the independent variables of age, plaque, gingival bleeding, healthy dentition, use of toothpicks and/or dental floss, intake of biscuits etc, soft drinks, and jam/honey, and the education of the child’s mother had statistically significant influence on median DMFS (p = 0.0001–0.02). However, only gingival bleeding after probing (OR = 1.2, CI = 1.0–1.5, p = 0.048) and the higher education level of the mothers (OR = 0.9, CI = 0.8–0.99, p = 0.03) significantly influenced the median DMFS in the multivariate analyses.

### DISCUSSION

This study provides information on dental caries status and caries-associated factors in a target group of 7–17-year-old children, covering schoolchildren from first to final grade (n = 1004). The total population of children in the target group in the central part of Moscow is 4000 children. However, all children (1004) in the examined school are included. The study was conducted in the Central District of Moscow, where the fluoride concentration in the drinking water is low (0.21 ppm). According to Kuzmina et al (2015), the district has lower caries experiences than other districts in Moscow.^11^

The population of the Central District of Moscow includes around 30,000 children aged 0–18. Schoolchildren are served healthy school meals, but they can also buy sweets. Socioeconomic status in the Central District of Moscow is higher than in other districts. Dental care for children is provided by the public dental health service (PDHS). The fully equipped clinics provide simple treatments, while the less-equipped clinics are mainly used for dental check-ups and screenings. Public dental clinics provide all types of treatments.

The target group was not randomised in the traditional way, as the children were enrolled as they came to the clinic, during the fixed examination period. The socioeconomic status of the parents varied within the different Moscow districts, so caution should be shown on generalising the results for the whole of Moscow.

The examinations took place at public schools in the Central District of Moscow, by four calibrated local dentists, and their reproducibility was substantial.^1^ Non-cavitated lesions were not included, nor were X-rays used, so the caries level is underestimated.^17^ Even though the registrations are comparable with statistics from Russia,^10^^–^^14^ the level of defs and DMFS in some ages is not logical in the present study (Table 2). This might be explained by selection bias.

The mean DMFS among 11–12-year-olds examined in the present study was 2.4, corresponding to a mean DMFT of 2.1 ± 0.2, which corresponds to the mean DMFT among the 12-year-olds in Moscow in 2013.^11^ The mean defs among 7–8-year-olds examined in the present study was 12.2, corresponding to deft of 1.6 ± 1.9. Data from Moscow on 6-year-olds in 2012 show a mean deft of 4.7.^14^

Bivariate analyses disclosed that 6 out of 11 variables were associated with levels of defs. Age was a major factor. When multivariate analyses were performed, only age, gender and the occurrence of plaque continued to play a statistically significant role for defs. Boys aged between 7 and 10, with presence of thick plaque had higher defs. Association between plaque score and caries prevalence in primary dentition was also reported by other authors.^2^

In permanent teeth, the bivariate analyses showed that nine variables influenced the DMFS. Again, age was a statistically significant factor, and sugar intake, such as biscuits, soft drinks and jam/honey also played a role in bivariate analyses. The multivariate analyses disclosed that only the level of the mother’s education and gingival bleeding continued to play statistically significant roles. This was in accordance with findings from other studies which showed that the mother’s education level was significantly associated with caries prevalence both in primary and permanent dentition.^16^

Knowledge of possible risk factors is of high importance on restructurings existing dental health service and planning caries preventive strategies. The present study indicates that caries in primary dentition is a major problem, while caries in permanent dentition in the age group included is of moderate severity. As the caries experience in primary dentition is very high at the age of 7–10 years, caries prevention must commence long before this. A modern caries control programme for primary dentition will implement dental health education focused on good quality of tooth cleaning. Parents shall be instructed to use toothpaste with fluoride, and parents should receive dietary advice. The fluoride concentration in the toothpaste must be at least 1000–1100 ppm from the first tooth’s appearance. As the fluoride concentration in the water supply in the district is rather low (0.21 ppm), dental fluorosis can be avoided by restricting the amount of toothpaste used each time, even if the toothpaste’s fluoride concentration is 1450 ppm.^6^

The education of children and parents in oral health is of high importance. There are a number of prevention programmes running at some schools for schoolchildren and the parents. The teachers could play a major role in oral health education and must be key motivators. Previously implemented prevention programmes at some schools in Moscow included teachers at schools and kindergartens as key motivators and showed good results. Nexo-study could be an example, as it shows a long-lasting effect of intensive oral health education received in childhood along with effect of non-operative treatment.^10^ There are experiences in running school programmes by university teachers, involving dental students, dental hygienists, all given lectures and training children according to age. At some schools, teachers continued these activities by small projects (eg, drawings done by children). So, there might be different ways to educate children and parents in oral healthcare. The results of the present study can be of importance to stress an attention on factors influencing caries development in different ages (eg, sweet milk among young children or soft drinks among older ones, etc).

Implementing supported projects could also be another way to improve the education; projects in association with World Oral Health Day supported by the FDI or local dental associations, or Bright Smile supported by Colgate, in which university teachers, dentists and hygienists go to schools and give lectures/lessons/training.

The question is when to initiate a programme. The youngest children included in the Greenlandic programme were around 1 year old. In Moscow, children should be invited to the PDHS at the age of 14 months; but in reality, many come to the dentist much later, at the age of 3 years. Before the PDHS in Moscow was restructured, there were dental clinics at the paediatric clinics, and young children were screened by dentists at an earlier age. But these dental clinics do not exist anymore and now parents come to the dentist when needed. Since in Russia paediatricians see children from the time where they are born, cooperation between the PDHS and paediatricians could be a great advantage for the child. Results of the recent questionnaire study showed that parents of young children need more oral health education, while the involvement of paediatricians in oral health education is rather limited. Systematic classroom-based health education is justified to enable children and parents to tackle the challenges in relation to control and prevention of caries risk factors. Involving the schoolteachers and dental staff may lead to a great impact in improvement of oral health education of children and parents. Greenland has been recognised as a country with a relatively high caries experience among children and adolescents. Greenland’s dental healthcare authorities wished to improve the situation and cooperation between dental school in Copenhagen and the Public Dental Service in Greenland was established in 2007, and a caries preventive programme covering children aged 0–15 was soon devised.^6^ The strategy was soon documented to be cost-effective, with a statistically significant caries reduction among 3- and 9-year-olds without any increase in costs.^6^

 Most mothers breastfeed their babies during the first year, but they do not brush their babies’ teeth and do not use toothpaste. Many paediatricians give advice to parents, mainly focusing on child nutrition, brushing teeth and choosing toothpaste. Only few paediatricians stress the importance of visiting the dentist when the first tooth erupts, but half of them recommend starting to brush the baby’s teeth at this point. They believed that ‘plaque’ and ‘food’ are factors in caries development, but only 20% of paediatricians think that ‘toothpaste’ is an important factor. As our results show a higher level of defs/DMFS with age, attention should also be focused on regular recalls, including examinations, diagnosis, training the children in toothbrushing, and dietary advice. The results from the present study may be used to investigate the cost effectiveness of a caries prevention strategy for children in Moscow.

## CONCLUSIONS

This study has identified clinical, socioeconomic and behavioural determinants for dental caries in primary and permanent dentition in school children in Moscow. In primary dentition, dietary advice must focus on reducing the intake of sweetened milk. In permanent dentition, attention must focus on reducing frequency of consumption of sweets. Oral health habits, the education of mothers and coordination with paediatricians may play a major role in efficient reduction of caries among young children. The findings can be used to improve oral health promotion for children and adolescents. Different ways to educate children and parents in oral health care is needed.
